# Predictability of the Retrieval Site Does Not Modulate Interference: Evidence From Reflexive Attraction

**DOI:** 10.1162/OPMI.a.275

**Published:** 2025-12-18

**Authors:** Maayan Keshev, Kaiva Hinkle, Matthew Wagers, Brian Dillon

**Affiliations:** Department of Linguistics, Hebrew University of Jerusalem, Jerusalem, Israel; Department of English, National Taiwan Normal University, Taipei, Taiwan; Department of Linguistics, University of California, Santa Cruz, Santa Cruz, CA, USA; Department of Linguistics, University of Massachusetts Amherst, Amherst, MA, USA

**Keywords:** prediction, agreement attraction, retrieval interference, reflexive pronouns

## Abstract

Syntactic dependency formation in comprehension is subject to retrieval interference that occurs when comprehenders need to activate stored information in memory to form and interpret a linguistic dependency. For example, retrieving a subject phrase to attach it to the verb might result in agreement attraction errors. It remains unclear whether this interference arises as part of routine dependency formation or as part of a repair mechanism that is activated when predictive dependency formation fails (e.g., Wagers et al., 2009). For example, it has been argued that reflexive anaphors resist attraction in comprehension because number/gender features of unpredictable elements are not associated with a strong ‘prediction error’ signal that might trigger retrieval-based and error-prone repair processes (Parker & Phillips, 2017). We test a version of the “Error-driven Retrieval” hypothesis by examining the interaction between reflexive attraction and the predictability of the anaphor. In two reading time experiments and one offline interpretation experiment, we find that the predictability of a reflexive dependency does not modulate its susceptibility to interference effects in comprehension. We propose that attraction is better captured as part of routine retrieval processes and that the (in)sensitivity of reflexives to structurally irrelevant distractors should be explained through other mechanisms.

## INTRODUCTION

One of the central tasks in sentence comprehension is dependency formation: identifying structural and interpretive links between different words and phrases. Dependency formation typically requires integration of information in the input across time. To execute this task, comprehenders use at least two types of processing mechanisms (Ferreira & Chantavarin, 2018)—forward and backward-looking. There is good evidence that comprehenders apply forward-looking parsing operations, such as predicting upcoming constituents of the sentence (Ferreira & Qiu, 2021; Kutas et al., 2011). Comprehenders also use backward-looking operations to construct linguistic dependencies, such as retrieving previous constituents from working memory when those are needed for a dependency. Much research has established that memory retrieval is subject to interference in general (McElree, 2006; Oberauer, 2019) and in sentence processing specifically (Lewis & Vasishth, 2005; McElree, 2006).

This picture raises questions about the trade-off between forward and backward-looking processing, and the extent to which routine processing relies on potentially interference-prone, backward-looking memory retrieval operations. In this study, we consider these questions in the context of agreement dependencies and reflexive anaphors, which have been a primary test case for memory interference in sentence processing. Specifically, we test the hypothesis that routine retrieval is not prone to interference, yet some interference-prone retrieval operations are deployed in response to prediction error, e.g., when the expected features of some predicted element conflict with those in the input. This hypothesis predicts that the susceptibility of any given dependency to retrieval interference should correlate with its predictability: The more a comprehender can predict the completion of a dependency, the greater the error signal that any unexpected input will generate, and hence, the more we should see retrieval interference. To preview our conclusions, we find that interference does not trade off with prediction in the processing of reflexive anaphors—we find evidence that retrieval interference for reflexive dependencies occurs in both predictable and unpredictable reflexives. We propose that backward-looking retrieval relies on error-prone memory mechanisms not uniquely linked to repair processes or prediction errors.

### Interference in Dependency Formation

Dependency formation that leans on memory retrieval may suffer adverse effects of similarity-based interference, leading to high processing time or to processing errors. Such hindered processing arises in various linguistic contexts, including dependencies between a verb and its subject (Cunnings & Sturt, 2018; Van Dyke, 2007; Wagers et al., 2009; among others), an ellipsis site and its antecedent (Martin & McElree, 2008, 2011; Parker, 2022), a possessive marker and its associated noun (Stone et al., 2021), a gap and its filler (Saul et al., 2025; Villata et al., 2018).

Interference that manifests in high reading times (inhibitory interference) depends on similarity of the target noun (e.g., the grammatical subject of a verb) and the distractor noun in structural position (e.g., subjects in clauses other than the one being processed; Van Dyke, 2007) and semantic contents (Van Dyke, 2007; Van Dyke & Lewis, 2003; Van Dyke & McElree, 2011). Based on the Cue-based Retrieval model (Lewis & Vasishth, 2005), it is assumed that inhibitory interference occurs when retrieval cues do not distinctly identify the target, and thus, cue overload slows down the retrieval of the fully matching item (see Jäger et al., 2017, for a review).

In addition to increasing processing time, interference can also increase the probability of forming an incorrect dependency. This can lead to a (temporary or rushed) acceptance of ungrammatical or incoherent sentences as grammatical. This arises when the target item does not match retrieval cues, yet a distractor presents a partial match to retrieval cues and leads to a “grammaticality illusion”. This type of interference is reflected in faster reading times of ungrammatical sentences, which otherwise would create processing difficulty and reading slowdown (facilitatory interference). The most well-studied case of grammaticality illusions or facilitatory interference is agreement attraction.

In agreement attraction, an agreement dependent (e.g., a verb) fails to match with the grammatically licensed agreement controller (**target**) because it instead matches with a grammatically unlicensed **distractor**. Two common instances of agreement attraction in English are exemplified in (2). In (2a), the verb *BE* does not agree with the singular DP “the door to the laboratories” but instead it agrees with the plural DP “the laboratories.” This occurs even though the distractor DP (underlined) is the complement of a preposition, and hence is not in a position to control agreement (the head which controls agreement is in bold). In (2b), the target verb *rely* agrees with the plural DP “the translators”, instead of the singular DP “the diplomat”. Here, the attractor is in the right kind of position to control agreement (i.e., a subject position), but it is not the subject of the relevant clause/verb. Remarkably, the verb seems to “skip” the immediately adjacent controller and agree with the further-away distractor. Both sentence types in (2) appear to be easily overlooked by comprehenders, as attested by reading time patterns, acceptability judgments, and ERPs (Deevy, 2000; Dillon et al., 2013; Pearlmutter et al., 1999; Tanner et al., 2014; Wagers et al., 2009).

(2)  *Two examples of agreement attraction in English subject-verb agreement*
   a. [The **door** to the laboratories] were accidentally left unlocked.
   b. The translators [who the **diplomat** rely on] worked furiously at the summit.

One theory of these attraction effects in comprehension attributes them to memory retrieval mechanisms. Such theories trace the error to the procedure by which the controller of agreement is identified. The most prominent theory is cue-based retrieval (Lewis & Vasishth, 2005), which explains the error as the consequence of using a content-addressable or associative memory to link syntactic dependents in real-time production and comprehension (Badecker & Kuminiak, 2007; Wagers et al., 2009). In this approach, upon encountering the verb, a backward retrieval for the subject is initiated. This retrieval is vulnerable to interference from distractors that match the verb’s features, and so can result in attraction errors—erroneous activation of a distractor.

### The Error-Driven Retrieval Model

In both reading times and acceptability judgments, the presence of an attractor exerts a larger influence on the perception of ungrammatical strings than it does on grammatical strings (the so-called *grammatical asymmetry*). (3) illustrates this asymmetry with rates of acceptance in a speeded grammaticality task (Wagers et al., 2009, Experiment 7): the presence or absence of a plural attractor in (3a) negligibly changes perceived acceptability in grammatical strings, but it has a large impact in ungrammatical strings like (3b).

(3)  a. *Grammatical string*      93%, 91%
     The **door** to the laboratory/laboratories **was** accidentally left unlocked.
   b. *Ungrammatical string*     25%, 55%
     The **door** to the laboratory/laboratories were accidentally left unlocked.

The grammaticality asymmetry has been widely replicated in reading time (Dillon et al., 2013) and ERP experiments (Tanner et al., 2014) and confirmed in a large-scale meta-analysis of reading studies (Jäger et al., 2017). On the face of it, this suggests that the attractor does not mistakenly lead the agreement computation astray very often in grammatical strings. If it had, comprehenders would be slower to access the target when a matching distractor is present (under a Cue-Based Retrieval account of attraction) or perceive the sentence as ungrammatical more often (under an encoding account of attraction).

One account for this asymmetry is that cue-based retrieval is triggered by prediction error, so we refer to it as the **Error-driven Retrieval Model** (Wagers et al., 2009). Wagers and colleagues proposed that the recognition of a subject in English allowed comprehenders to predict the morphological features (e.g., grammatical number) of an upcoming verb. These predicted features are then checked against the contents of the new input. In grammatical sentences like (3a), this prediction is satisfied, and comprehension proceeds smoothly (either without retrieval—Tanner et al., 2014; or with a ‘routine’ retrieval process that is highly constrained by structural cues—Parker & Phillips, 2017). However, in ungrammatical sentences like (3b), the prediction is not met, triggering an error. On the Error-Driven retrieval view, this error occasions a cue-based retrieval in an attempt to retrieve the agreement controller based on cues provided by the verb. Because these cues include the plural number feature of the verb, they will resonate with the attractor, creating interference from the attractor selectively in the ungrammatical strings (3b).

Support for this hypothesis comes from the observation of a divergence between the timing of a simple grammaticality effect and the timing of an effect of attraction in ungrammatical strings. Lago et al. (2015) demonstrated this for both Spanish and English comprehenders using a distributional analysis of reading times (RTs) in self-paced reading. In all four of their experiments, attraction emerged only when participants were reading the word immediately following the verb. This may seem unsurprising for self-paced reading, where effects are often observed first at the word or phrase immediately following the critical region (i.e., “spill-over”). But, in two of their four experiments, a simple effect of grammaticality was found on the verb itself, preceding the attraction effect. Lago et al. (2015) analyzed the shape of the RT distribution at this spillover region using Vincentile analysis (Ratcliff, 1979; Staub, 2010; Vincent, 1912). There, they found that grammaticality affected the earliest RT vincentiles—effectively shifting the entire RT distribution—whereas attraction affected the latest RT vincentiles. One interpretation of these findings is that attraction only affects a small number of influential trials in ungrammatical conditions, located in the tail of the RT distribution (Staub, 2009, 2010). This is congenial to a two-stage process, according to which grammaticality can be registered at an early stage via prediction error. Only later does the presence of a plural attractor interfere. It does not, however, uniquely implicate such a model. For example, it may be that the effect of the attractor is present in many trials, but simply has not accrued enough strength to affect the earliest vincentiles of the RT distribution. The apparent staging of these effects—grammaticality before attraction—is consistent with the Error-Driven retrieval model’s hypothesized time course of processing.

It should be noted that the Error-based Retrieval model does not claim that retrieval only arises in processing as a product of a prediction error. Routine retrieval, which is not triggered by disconfirmed predictions, can arise in the context of unpredictable dependencies. That is, if a dependency was not formed predictively, then successful dependency completion would presumably involve backward-looking retrieval; previous parts of the linguistic context would not be otherwise accessible for dependency formation. It is also theoretically possible, on this view, that routine retrieval arises for some predictable dependencies. It could be that an early dependent initiates a forward prediction for the right edge of a dependency, but with only a subset of the features actively predicted (Wagers & Phillips, 2014). For example, the comprehender might predict syntactic subcategorization features of the later dependent but not any lexico-semantic features. In that case, even if the syntactic prediction was confirmed, retrieval might still be necessary to allow for the integration of the unpredicted lexico-semantic features (see Wagers & McElree, 2013; Wagers & Phillips, 2014, for discussion).

### The Role of Predictability in Error-Driven Retrieval

Prediction in sentence processing can apply to various types of linguistic representations. Comprehenders generate expectations about specific lexical items (Ito et al., 2020; Kutas et al., 2011; Ness & Meltzer-Asscher, 2018) as well as about the structure of upcoming input (Hale, 2001; Levy, 2008; Staub & Clifton, 2006). As formulated in Wagers et al. (2009), the Error-driven Retrieval model relies on the latter type of predictability—the prediction of grammatical dependencies, specifically the relationship between a subject and a verb. Lexical input or its syntactic structure can be unpredictable either because the context is low-constraining or because the context strongly predicts a different continuation. The Error-driven Retrieval model assumes a syntactically constraining context, where the comprehender anticipates a dependent (along with its specific grammatical features). Under these conditions, a forward-looking search for a grammatically appropriate dependent can be initiated. Presumably, verbs are predictable (later) dependents in such a view since they are generally grammatically required once a subject phrase has been identified. If comprehenders routinely anticipate verbs, then their agreement features can be predicted ahead of time as well. To the extent that any later verb mismatches predicted features, this constitutes disconfirmation of a prediction, which we label here *prediction error*.

Yet, empirically, retrieval interference does not uniquely arise in such grammatically constraining contexts. Retrieval interference has also been observed for dependencies that are presumably less predictable. For example, interference has been observed with reflexive pronouns (Jäger et al., 2020; Parker & Phillips, 2017), adjunct control (Parker et al., 2015), and ellipsis (Martin, 2018; Parker, 2022), among others. While interference has been observed in presumably unpredictable dependencies, it has been argued that attraction effects for some of these dependencies are diminished relative to agreement dependencies. More specifically, reflexive anaphors have been claimed to partly resist attraction (Dillon et al., 2013; Parker & Phillips, 2017; but cf. Jäger et al., 2020). This raises the question of whether routine retrieval, which is not triggered by disconfirmed prediction of agreement features, is vulnerable to retrieval interference to a similar degree as retrieval associated with a prediction error. Since the predictability of those dependencies was not quantified or directly manipulated, the above studies do not settle the question.

To examine how predictability interacts with retrieval interference in comprehension, a direct comparison of predictable and unpredictable dependencies is required. Because verbs and their morphological forms are generally predictable, verbal agreement dependencies do not provide a fitting test case. In the next section, we turn to reflexive anaphors, which can realize a wider range of predictability and which are prone to attraction, although in a narrower range of circumstances. In the next section, we also review the debate around reflexive attraction and discuss how accounts of reflexive attraction (or lack thereof) posit a tradeoff between predictability and retrieval interference in line with the Error-driven Retrieval model.

### Reflexive Attraction as a Case Study

The Error-driven Retrieval model makes the following key prediction: Attraction would be avoided, or at least reduced, when later dependents are less predictable. If comprehenders do not generate strong predictions for an upcoming dependent, then the likelihood that its morpho-syntactic features clash with predictions is low. The diminished rate or magnitude of the syntactic prediction error would mean that error-prone memory retrieval is less likely to be triggered as well.

As mentioned above, it is challenging to test the interplay between dependency prediction and agreement attraction with verbal agreement features. English speakers appear to routinely generate expectations for verbs with certain agreement features even in contexts where those features are not ultimately morphologically realized (Solomon & Pearlmutter, 2005). However, it is straightforward to manipulate the predictability of another agreeing dependent, which is widely studied in the attraction literature—direct object reflexives in English. Consider (4):

(4)  a. The host scorned himself.     Pr(himself|scorned) = ∼0.00
    b. The host disgraced himself.    Pr(himself|disgraced) = 0.25

The presence of a reflexive in (4a) is highly unlikely: A search of the COCA corpus (see Materials below) failed to find any occurrences of a reflexive following *scorned* (out of 648 tokens). In contrast, there is a one-in-four chance that a reflexive will follow *disgraced* in a sentence like (4b). Comprehenders should therefore have a greater expectation for a reflexive after *disgraced* than after *scorned*. This is therefore one place to test the hypothesized relationship between predictability and attraction effects. Manipulating the lexical predictability of a reflexive anaphor can trigger predictive dependency formation, with a prediction of specific syntactic features. This would allow a comparison of violations that trigger prediction errors to a greater or lesser extent (high predictability reflexives vs. low predictability reflexives, correspondingly).

One challenge for this test concerns the availability of predictive dependency formation in those cases. Manipulating how surprising a reflexive is in a given verb does not necessarily modulate whether the parser predicted it as part of an agreement dependency initiated by the antecedent. We see this manipulation as a reasonable starting point for investigating the assumption of the Error-driven Retrieval model. We assume that the high lexical predictability of the reflexive leads prediction of the reflexive with its agreement features, and thus, in effect, triggers formal agreement computation. In that sense, this predictability contrast puts the Error-driven Retrieval model to the test. Still, this assumption can be debated, and an Error-driven Retrieval model, which strongly differentiates contextual predictability from predictive dependency formation, might not be implicated by such reflexive predictability. We therefore formulate our predictions in terms of a general version of the Error-driven Retrieval model and return to this in the discussion.

Another challenge for this test is the late initiation of the prediction for a dependency. The error-driven retrieval account builds on the assumption that agreement features are predicted by the agreement controller (the subject). The manipulation in (4) does not modulate the predictability of the dependency at the subject, but later on, at the verb. Classically, the prediction of the upcoming dependent should arise at the. However, we assume that initiation of the prediction after the controller and before the dependent would produce similar results since the features of the controller would either still be active or retrieved successfully (routine retrieval). Still, we treat this as testing for a general version of the Error-driven Retrieval approach, since some versions might not follow these assumptions. In the general discussion, we return to a possible interpretation of our study if one does not accept the premise above.

Reflexive anaphors also present an interesting test case since the ideas rooted in the Error-driven Retrieval have featured in the debate over whether reflexives are subject to retrieval interference or attraction. Sturt (2003) and later Dillon et al. (2013) argued that they were not, at least in early processing measures, based on a failure to find evidence of interference from grammatically inaccessible antecedents. This finding was corroborated by several other studies (Cunnings & Sturt, 2014; Kush & Phillips, 2014) and a meta-analysis in Jäger et al. (2017). However, more recent work suggests that reflexives are susceptible to attraction effects in some contexts: Jäger et al. (2020) argue that attraction can be observed with a sufficiently large participant sample; Parker and Phillips (2017) find evidence for attraction effects when the reflexive mismatches its local subject in more than one agreement feature (see also Sloggett, 2017); and Yadav et al. (2022) show this effect is present but highly variable across individuals, with only a subset of individuals in an experiment showing reflexive attraction (see also Cunnings & Felser, 2013). In general, it seems that reflexive dependencies are subject to attraction-like interference in some contexts, but the precise factors that make reflexive dependencies susceptible to attraction remain unclear.

An important question is why reflexives (partly) resist attraction. Parker and Phillips (2017) invoke ideas rooted in the Error-driven Retrieval model to explain this observation. The unpredictability of reflexive dependencies means that to be integrated with their antecedent (and receive reference), reflexives have to initiate a backward-looking retrieval. In addition, the reflexives’ unpredictability also means that there is less likely to be a set of predicted features that the reflexive’s features would conflict with. If reflexives are unlikely to generate the type of prediction error that the Error-driven Retrieval model invokes, and an antecedent has to be retrieved, the conclusion is that retrieval of a reflexive’s antecedent is part of routine reflexive processing, rather than an error-driven retrieval (Dillon, 2014). Parker and Phillips (2017) suggest that when retrieval is part of routine processing (i.e., not triggered by a prediction error), comprehenders may upweight structural retrieval cues. By strongly weighting structural constraints, distractors will compete less at retrieval, making reflexive anaphors (and other unpredictable dependencies) less vulnerable to attraction.

More specifically, Parker and Phillips (2017) argue that comprehenders particularly downweight structural cues whenever a retrieval is triggered by a prediction error because that error signal undermines the comprehender’s confidence in the utility of the structural cues. This explains why predictable dependencies like agreement should appear relatively unconstrained by hierarchical structure when such retrievals are triggered. On the other hand, any retrievals involved in resolving the reflexive reference should weigh structural cues quite highly, minimizing the impact of distractors that are merely feature-matched. Under this proposal, the reflexives’ robustness against attraction is another reflection of the trade-off between prediction and retrieval in dependency formation: The more predictable a dependency is, the more vulnerable it would be to retrieval interference in ungrammatical sentences. This makes reflexive attraction a particularly interesting test case for the Error-driven Retrieval proposal.

Overall, in this study, we test the predictions of the Error-driven Retrieval model’s general proposal by testing how the predictability of a reflexive anaphor affects. This aims to test the proposal that reflexive attraction is more constrained than verbal agreement attraction due to the relatively low predictability of the dependent element. Across three experiments, we manipulate the predictability of the reflexive given the verb and measure rates of reflexive attraction in reading times and interpretation tasks. If structurally insensitive retrieval processes are linked to prediction error in the way implied by the Error-driven Retrieval model, and the features of the subject are accessible at the point of the verb (as implied by the general version of the cue-based retrieval model), then retrieval interference for reflexive dependencies should be larger when the occurrence of a reflexive is predicted. This should arise because if the comprehender can predict a reflexive and predictively compute its features based on the subject, there is some chance the predicted reflexive would mismatch the predicted form in the input and hence could trigger an interference-prone repair retrieval operation.

## EXPERIMENT 1: ONE-FEATURE MISMATCH ATTRACTION

### Methods

#### Participants.

We recruited 120 self-reported native English-speaking participants through the Prolific Academic online platform. Participants gave informed consent and received monetary compensation of 4$ (a rate of approximately 7$/hr).

#### Materials.

We operationalize the reflexive’s predictability as the probability of a reflexive pronoun given the verb, under the assumption that using verbs that are usually associated with reflexive activities and followed by pronouns would make a reflexive pronoun predictable. We constructed 24 item sets of six conditions. In a 2 × 3 design, we manipulated the reflexive’s predictability given the verb (high vs. low predictability) and the availability of a noun matching the reflexive (target-match, distractor-match, and no-match). The experimental items were distributed across six Latin Square lists. Each list was combined with the same set of 48 filler items. All filler items were grammatical, including 12 sentences of a structure similar to those of experimental items with grammatical reflexive anaphors.

To manipulate the reflexive’s predictability, frequency information was collected from the Corpus of Contemporary American English—COCA (Davies, 2008). An initial set of verbs that commonly depict reflexive events was collected from Levin (1993). The other initial verb set, for which reflexives would be highly unpredictable, was collected from Bergeton (2004). Then, the probability of a reflexive anaphor immediately following each verb (of both categories) was calculated. From each verb category, we selected verbs based on overall frequency (to ensure participants are familiar with the verb and its statistics) and on extreme rates of collocation with a reflexive (selecting verbs with the highest and lowest collocation rates). The average probability of a reflexive given the verb was 0.01 in the low predictability conditions and 0.29 in the high predictability conditions (across sets). This manipulation created sets with an average difference of 5.01 bits of Surprisal in the reflexive’s predictability. As verbs that are highly predictive of a reflexive are hard to find, low predictability and high predictability conditions also differed in verb frequency. The average frequency of verbs predictable of a reflexive was 1,231 occurrences, while for the other set of verbs, the average frequency was 14,825 occurrences.

The materials included both masculine and feminine reflexive forms across different sets. Within each set, we kept the reflexive form constant while shifting the form of the target subject (from feminine to masculine or vice versa) to create the ungrammatical conditions (Distractor match and No match). To make sure that the subject cannot be a grammatical antecedent of the reflexive in those ungrammatical conditions, which function as the attraction contrast, we used nouns with strict grammatical gender (e.g., *hostess* in Table 1) rather than stereotypical gender (e.g., *nurse*). Lastly, due to the sparsity of grammatical gender in English nouns, distractor gender was manipulated using the stereotypical gender of proper nouns (e.g., *Wendy* vs. *Ethan* in Table 1).

**Table 1.** Example of an experimental item set from Experiment 1. The critical reflexive is in bold, and the competing antecedents (the target and the distractor) are underlined. Bracketed verbs represent low and high predictability correspondingly.

Feature matching	Sentence
Target match	The host who worked with Wendy {scorned | disgraced} **himself** recently for arriving extremely late.
Distractor match	The hostess who worked with Ethan {scorned | disgraced} **himself** recently for arriving extremely late.
No match	The hostess who worked with Wendy {scorned | disgraced} **himself** recently for arriving extremely late.

In most sets (20 out of 24), both variants of the target noun (in the grammatical and the ungrammatical conditions) had strict grammatical gender (e.g., *father* and *mother*, *bride* and *groom*, *nephew* and *niece*). However, in a minority of the sets (4 out of 24), the masculine variant used could be interpreted as gender neutral (e.g., *host* in Table 1, *waiter*, *actor*). Even if those nouns indeed are grammatically gender neutral, they are likely to be assigned the masculine gender. The availability of an alternative that would be used if a feminine referent were intended should bias readers toward the masculine reading. Therefore, in those sets, a masculine reflexive pronoun was considered grammatical. Still, with those nouns, a feminine reflexive might not be strictly speaking ungrammatical. Consequently, those potentially gender-neutral nouns were only used in the grammatical baseline condition (Target match), to avoid a potential “grammatical” reading of the conditions in the attraction contrast (No match and Distractor match).

#### Procedure.

The experiment was implemented as a Maze task (Boyce et al., 2020; Forster et al., 2009; Freedman & Forster, 1985). In this task, participants are asked to repeatedly choose the next word of the sentence by deciding between two words presented on the screen. Competitor words in this experiment were real words that did not fit in the sentential context. To reject the competitor words and choose the correct word, participants have to integrate the structure and the meaning of the sentence so far. The time to selection is treated in this task as a measure of the processing difficulty comparable to reading time in word-by-word self-paced reading (Boyce et al., 2020; Boyce & Levy, 2023). Moreover, the reaction time (RT) measure in the Maze task allows a highly localized approximation of processing difficulty as selection requires comprehensive integration of the correct continuation with the previous context.

The maze task was used to detect incremental processing operations in various contexts including attachment ambiguities (Boyce et al., 2020; Freedman & Forster, 1985), the subject/object gap asymmetry of relative clauses (Forster et al., 2009; Witzel & Witzel, 2021), predictability (Boyce & Levy, 2023; Husband, 2022), and interference (Fujita & Vasishth, 2024; Fujita & Yoshida, 2024). Therefore, we assume that this task can detect the costs of retrieval processes involved in sentence comprehension.

The experiment was implemented in PCIbex—PennController for Internet-Based Experiments (Zehr & Schwarz, 2018). Maze competitors were generated automatically using the A-Maze generation process (Boyce et al., 2020) and were consistent across different conditions of the same set. We used the error correction variant of the Maze task (Boyce & Levy, 2023). In this variant, participants receive an error message and are forced to re-select the correct option if they have made a mistake. This ensures that participants are discouraged from guessing since providing wrong responses extends the duration of the experiment.

Participants performed the experiment remotely on their computers. Participants were randomly assigned to a list. Before starting the experiment, participants undertook a practice block of five sentences. No comprehension questions were featured in the experiment—the only task was choosing the word that correctly continued the sentence. The experiment took approximately 35 minutes.

#### Data Analysis.

Participants were excluded from the analysis if they failed to choose the correct Maze alternative in more than 5% of the trials (resulting in the removal of 15 participants). In addition, we excluded from analysis data points from wrong responses and RTs that were either lower than 200 ms or greater than 3500 ms (affecting altogether 2.8% of the overall data).

We used sum-coding for a reflexive’s predictability given the verb (½ for low predictability and −½ for high predictability) and Helmert coding for the feature matching factor (Schad et al., 2020). The Helmert coding produced two predictors: Grammaticality, contrasting the target-match condition (−⅔) with the mean of the distractor-match and no-match conditions (⅓ each); and attraction, contrasting the distractor-match (−½) and the no-match (½) conditions. The models included a maximal random effect structure by-item and by-participant, including random intercepts and random slopes for all fixed-effects predictors.

As a post-hoc analysis, we also coded predictability as a continuous factor. Based on Surprisal (Levy, 2008), we used log(P(reflexive|verb)) for this analysis. We used the COCA frequency information that was extracted in the construction of the items (see subsection Materials). This analysis yielded similar posterior distributions and is available in Appendix B.

The RT data were analyzed in R (R Development Core Team, 2015) using Bayesian hierarchical models with a lognormal link function. We analyzed RTs at the critical reflexive region and on the following word (the spillover region), in separate models. We fitted Bayesian hierarchical models in Stan (Carpenter et al., 2017), via the brms package (Bürkner, 2017). Four Monte Carlo Markov Chains of 20,000 iterations each were sampled from the posterior distribution. The first 4,000 samples of each chain were discarded as a warm-up. Convergence was checked using the R-hat statistic, which was at 1.0 for all fixed effects.

**Figure 1.**
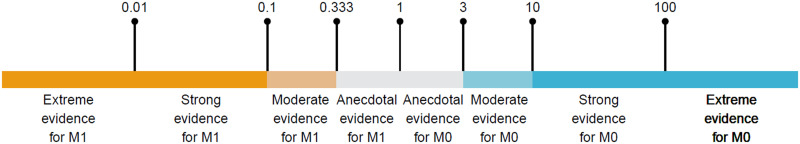
Interpretation guidelines for BF01.

We conducted Bayes Factor (BF) analyses to evaluate the evidence for the effects in question. The BF is a ratio of the data’s likelihood under different models. We use this to compare a model that includes all main effects and interactions (the hypothesis model) and a model where one of these predictors is completely removed, both from the fixed effects and from the random slopes (the null hypothesis). BFs were computed using the bridgesampling R package (Gronau et al., 2020). We follow the common guidelines for the interpretation of BFs (Lee & Wagenmakers, 2014), as illustrated in Figure 1.

As BFs are sensitive to the prior distribution (Gelman et al., 2017), we computed BFs for a range of plausible priors. Our set of weakly informative priors included a standard normal distribution, N(0, 1), as the prior for fixed effects and for the standard deviation parameters, a wide normal prior of N(0, 10) for the intercept, and the LKJ prior for correlation matrices of random effects (Lewandowski et al., 2009). Weakly informative priors on the fixed effect of interest (such as our N(0, 1) prior) are likely biasing the results for evidence for the null since much of the probability mass of the model is distributed over parameter values that are very different from estimates derived from the data (Mulder & Wagenmakers, 2016; Rouder et al., 2018). Therefore, for each effect of interest, we created models with more informative priors by decreasing the standard deviation of the prior associated with that effect to N(0, 0.5) and N(0, 0.2). This reflects the intuition that effects are expected to be rather small and concentrates more of the probability mass around more likely effect sizes. Priors associated with other fixed effects and with the random effects were kept identical.

### Results

**Figure 2.**
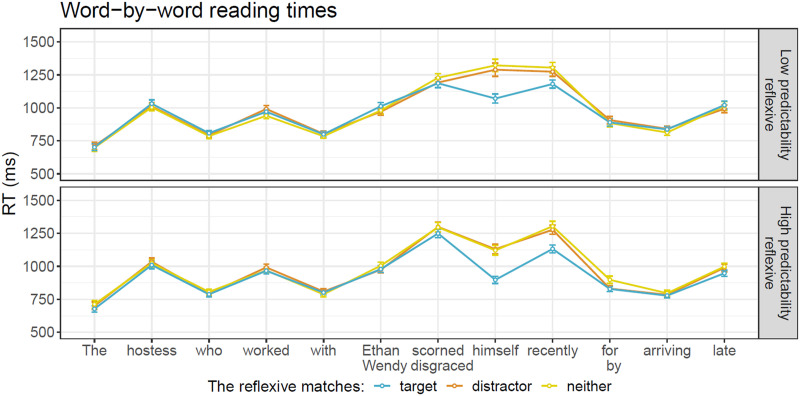
Average RT across regions of test sentences in Experiment 1. Error bars represent the standard error of the mean over participants.

**Figure 3.**
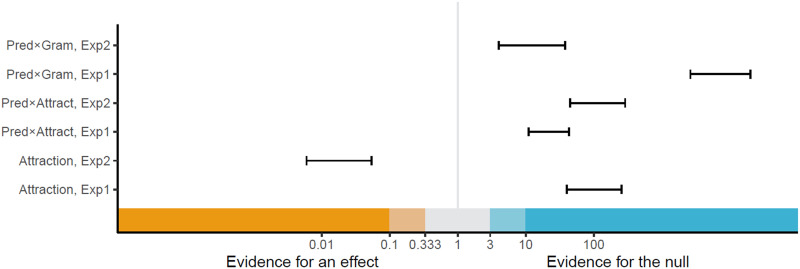
The range of Bayes factors for effects of interest in Experiment 1 and Experiment 2. Effects illustrated here correspond to those in the reflexive region column of Tables 2 and 4.

Word-by-word RTs by condition are presented in Figure 2. The results of the different models are summarized in Table 2. Figure 3 summarizes Bayes Factor results across Experiments 1 and 2.

**Table 2.**
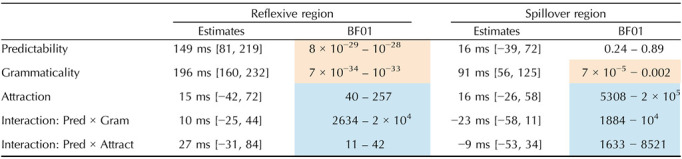
Results of Experiment 1. Mean and 95% credible interval of the posterior distribution for fixed effects (under the weakly informative priors set), as well as the range of BF evidence (across prior sets) for the fixed effects. Strong or extreme evidence for the null is shaded blue. Strong or extreme evidence for an effect is shaded orange.

The results of the BF analysis suggest extreme evidence for a grammaticality effect, such that RTs are slower in ungrammatical sentences (distractor-match and no-match conditions) relative to grammatical ones (target-match conditions), in both the critical region and the spillover region. The results also suggest extreme evidence for an effect of predictability at the reflexive (the critical region), such that unpredictable reflexives (following anti-reflexive verbs) trigger slower RTs than predictable ones (following pro-reflexive verbs). However, in both the critical region and the spillover region, we observe evidence against the attraction effect and against an interaction of attraction with predictability. We obtain strong to extreme evidence for the null hypothesis on the BF analysis of those factors. Lastly, we find extreme evidence against an interaction of grammaticality with predictability.

After collecting the data and reviewing our experimental items again, we wanted to examine whether the results in the low predictability conditions were biased by a subset of the items in which a reflexive pronoun might be incongruent, rather than just unlikely. When removing this subset of items from the low predictability conditions, the pattern remained the same (see Appendix C).

### Discussion

Experiment 1 suggests strong evidence against an interaction of reflexive attraction and predictability. Neither low nor high predictability reflexives exhibited attraction in this experiment. The insensitivity of attraction to the predictability of the reflexive (given the verb) speaks against the idea that retrieval interference reflects a repair mechanism. If interference-prone retrieval were triggered by a mismatch between the predicted dependent and the input, this retrieval should have been triggered for reflexives that are highly predictable given the verb, and attraction should be observed in those conditions. The finding that the dependent’s predictability is not a sufficient condition for attraction suggests that this is not the key difference between verbal agreement and reflexive attraction.

Experiment 1 also exhibited that the processing cost associated with ungrammaticality does not interact with predictability. This conflicts with the basic assumption of the repair notion: The Error-driven Retrieval proposal argues that when the retrieval site is predictable, ungrammaticality is accompanied by a more prominent prediction error; this prediction-error assumption also entails that ungrammaticality would be perceived as more severe where the ungrammatical constituent is contextually predictable; however, our results suggest that ungrammaticality of the reflexive disrupts processing to the same degree whether the anaphor is predictable or very surprising. If indeed processing of ungrammaticality is similar for predictable and surprising ungrammatical elements, retrieval procedures cannot be triggered or changed qualitatively based on the predictability of this ungrammatical element. Therefore, this finding conflicts with a key underlying assumption of the general Error-driven Retrieval proposal.

Still, Experiment 1 only suggests that a prediction error is not sufficient to yield attraction. This does not entail that when attraction arises, it is not a reflection of prediction error. It could be that other conditions are required to give rise to attraction, and that once those are met, the resulting attraction would be modulated by prediction error. For example, it could be that our use of the Maze task (a task that is very different from natural reading) blocks attraction from arising in our dataset.

To target this issue and extend the findings of Experiment 1, we conducted a second experiment that aimed to trigger attraction using the two-feature mismatch design (Parker & Phillips, 2017). Parker and Phillips (2017) found that when the target subject mismatches the reflexive in both number and gender, competition with the distractor arises and attraction effects are revealed. We accordingly amended our design to facilitate attraction. If attraction can arise in the Maze task, we would expect to find it under this two-feature design. Crucially, in Experiment 2, we not only replicate the two-feature attraction in the Maze task but also re-examine whether attraction interacts with predictability in this case.

## EXPERIMENT 2: TWO-FEATURE MISMATCH ATTRACTION

### Methods

#### Participants.

We recruited 120 self-reported native English-speaking participants through the Prolific Academic online platform. Participants gave informed consent and received monetary compensation of 7$ (a rate of approximately 12$/hr).

#### Materials.

Materials were based on those used in Experiment 1. We edited the changed subject noun from singular to plural. Occasionally, the post-critical adjunct was also edited to be semantically compatible with a plural subject. Other than that, experimental and filler items were identical to those of Experiment 1 (see Table 3).

**Table 3.** Example of an experimental item set from Experiment 2. The critical reflexive is in bold, and the competing antecedents (the target and the distractor) are underlined. Bracketed verbs represent low and high predictability correspondingly.

Feature matching	Sentence
Target match	The host who worked with Wendy {scorned | disgraced} **himself** recently for arriving extremely late.
Distractor match	The hostesses who worked with Ethan {scorned | disgraced} **himself** recently for arriving extremely late.
No match	The hostesses who worked with Wendy {scorned | disgraced} **himself** recently for arriving extremely late.

#### Procedure.

The experimental procedure was the same as in Experiment 1.

#### Data Analysis.

All statistical analysis procedures, including exclusion criteria, were identical to Experiment 1. Exclusion criteria resulted in the exclusion of 17 participants and affected 3% of the data of the remaining participants.

### Results

**Figure 4.**
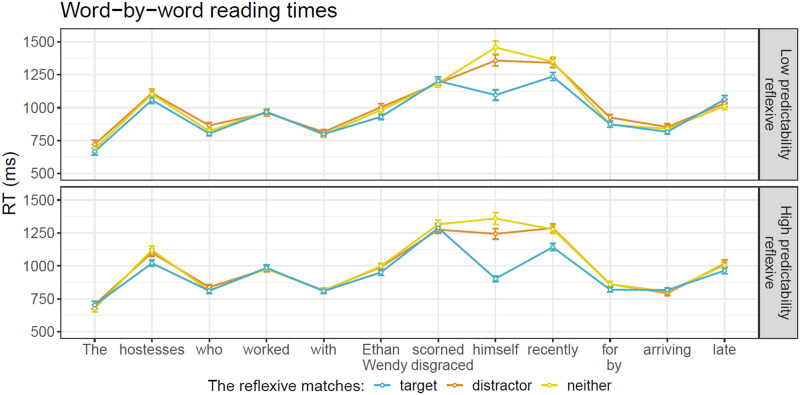
Average RT across regions of test sentences in Experiment 2. Error bars represent the standard error of the mean over participants.

Word-by-word RTs by condition are presented in Figure 4. The results of the different models are summarized in Table 4.

**Table 4.**
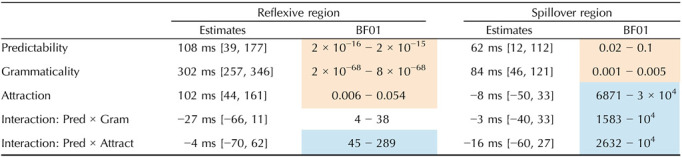
Results of Experiment 2. Mean and 95% credible interval of the posterior distribution for fixed effects (under the weakly informative priors set), as well as the range of BF evidence (across prior sets) for the fixed effects. Strong or extreme evidence for the null is shaded blue. Strong or extreme evidence for an effect is shaded orange.

The results of the BF analysis suggest extreme evidence for a grammaticality effect, such that RTs are slower in ungrammatical sentences (distractor-match and no-match conditions) relative to grammatical ones (target-match conditions), in both the critical region and the spillover region. The results also suggest extreme evidence for an effect of predictability at the reflexive (the critical region), such that unpredictable reflexives (following anti-reflexive verbs) trigger slower RTs than predictable ones (following pro-reflexive verbs). Crucially, we obtain strong to extreme evidence for an attraction effect in the critical region. However, this attraction effect was not modulated by predictability: we obtained strong to extreme evidence for a null interaction between attraction and predictability in this region. Lastly, we find moderate to strong evidence against an interaction of grammaticality with predictability, in the critical region, and extreme evidence against it in the spillover region.

### Discussion

Experiment 2 suggests evidence for reflexive attraction, but still gives rise to strong evidence against an interaction of it with the predictability of a reflexive given the verb. These findings suggest that the results of Experiment 1 are not likely to be due to some insensitivity of the Maze task to detect interference effects in general. In contrast to predictions of the general Error-driven Retrieval proposal, the predictability of the reflexive anaphor did not strengthen the attraction effect observed in this experiment. This provides additional evidence against the notion that retrieval procedures are more vulnerable to interference following a prediction error. In this experiment, evidence against the interaction of reflexive predictability and grammaticality was less strong (at the critical region, where attraction was observed). However, the experiment still provides moderate evidence against such interaction, conflicting with the assumptions of the general Error-driven Retrieval proposal.

It should be mentioned that concluding these findings as to the Error-driven Retrieval model depends on accepting the assumptions that (i) manipulating the lexical predictability of the reflexive generates predictive dependency formation, and that (ii) predictive dependency formation has similar properties when it is initiated at the agreement controller and when it arises after the controller and before the dependent. Thus, these results might speak against only some versions of the Error-driven Retrieval model. Overall, the results of Experiment 2 replicate and extend those of Experiment 1, in providing additional evidence against a version of the Error-driven Retrieval model.

## EXPERIMENT 3: COMPREHENSION ACCURACY

To further examine whether the adverse effects of interference are affected by our predictability manipulation, we conducted a final interpretation study. We speculated that an attraction manipulation could elicit stronger effects in accuracy measures, relative to the RT data. In Experiment 3, we, therefore, examined whether comprehenders interpret a distractor as the antecedent of the reflexive in attraction configurations and how this may interact with the predictability of the reflexive pronoun.

### Methods

#### Participants.

We recruited 48 self-reported native English-speaking participants through the Prolific Academic online platform. Participants gave informed consent and received monetary compensation of $3 (a rate of approximately $12/hr).

#### Materials.

We amended the sets from Experiments 1–2. First, in this experiment, we included a completely grammatical interference condition. Interpreting the distractor as the antecedent of the reflexive could reflect a late conscious process of extrapolation from stimuli perceived as ungrammatical. Therefore, we wanted to include another measure of potential interpretation errors, which should not reflect such strategic reasoning. In this new condition, both the target and the distractor matched the reflexive. Second, we removed the ungrammatical baseline condition (the no-match condition) to reduce the number of overall ungrammatical stimuli in the experiment and encourage non-strategic processing. The amended 2 × 3 design (see Table 5) manipulated the predictability of the reflexive given the verb (high vs. low predictability) and the availability of a noun matching the reflexive (target-match, distractor-match, and both-match). We treated the target-match condition as the baseline for the two other conditions.

**Table 5.** Example of an experimental item set from Experiment 3. The critical reflexive is in bold, and the competing antecedents (the target and the distractor) are underlined. Bracketed verbs represent low and high predictability, respectively.

Feature matching	Sentence	Response alternatives
Target match	The host who worked with Wendy {scorned | disgraced} **himself**.	The host, Wendy
Distractor match	The hostess who worked with Ethan {scorned | disgraced} **himself**.	The hostess, Ethan
Both match	The host who worked with Ethan {scorned | disgraced} **himself**.	The host, Ethan
Question: Who was {scorned | disgraced}?		

In addition, to make sentence comprehension simpler, we removed the post-reflexive material from the items. This change resulted in the exclusion of four verbs from the original list since they required additional arguments. These verbs were replaced with other similarly biased verbs (three reflexive-biased and one anti-reflexive) based on a COCA search. In these amended sets, the average Surprisal of a reflexive given the verb was 2.52 bits in the high predictability conditions (corresponding to an average probability of 0.30) and 9.23 bits in the low predictability conditions (corresponding to an average probability of 0.01).

The experimental items were combined with the same set of 60 filler items. All filler items were grammatical, and were designed to balance several factors: The proportion of pronouns v. reflexive anaphors in the experiment; the proportion of questions to which the correct answer was the first v the second noun in the sentence; and the proportion of questions to which the correct answer was a proper noun v. a common noun. The gender of the reflexive anaphor or pronoun was balanced between masculine and feminine across both experimental items and fillers.

#### Procedure.

The experiment was implemented as a rapid reading task. In this task, participants are asked to choose the correct antecedent to a reflexive anaphor by answering comprehension questions about sentences presented on the screen. The sentences were presented one word at a time for a fixed short time interval—each word appeared on screen for 250 ms with an inter-stimulus interval of 150 ms. The questions were presented after their respective sentences. Participants were then asked to choose either the target or the distractor as the antecedent to the reflexive anaphor. The experiment was implemented in PCIbex (Zehr & Schwarz, 2018). Participants performed the experiment remotely on their computers. Participants were randomly assigned to a list. Before starting the experiment, participants undertook a practice block of five sentences. The experiment took approximately 15 minutes.

#### Data Analysis.

Participants had an average accuracy of 87% for filler sentences. In addition, due to an experimenter error, one of the items did not conform to the design and was excluded from the analysis.

We analyzed accuracy using Bayesian hierarchical models with a Bernoulli link function. We used sum-coding for the reflexive’s predictability given the verb (½ for low predictability and −½ for high predictability) and repeated contrasts for the feature matching factor (Schad et al., 2020). The repeated contrasts’ coding produced two predictors: one contrasting the target-match condition with the double-match condition, and one contrasting the target-match condition with the distractor-match condition. The models included a maximal random effect structure by-item and by-participants, including random intercepts and random slopes for all fixed-effects predictors.

As in Experiments 1–2, we conducted a BF analysis with increasingly informative priors. Our set of weakly informative priors included a standard normal distribution, N(0, 1), as the prior for fixed effects and for the standard deviation parameters, a wide normal prior of N(0, 3) for the intercept, and the LKJ prior for correlation matrices of random effects (Lewandowski et al., 2009). For each effect of interest, we created models with more informative priors by decreasing the standard deviation of the prior associated with that effect to N(0, 0.5) and N(0, 0.2), while keeping priors of other fixed effects and with the random effects weakly informative. We sampled from the posterior distribution with four Monte Carlo Markov Chains of 10,000 iterations each. The first 2,000 samples were discarded as warm-up samples. Convergence was checked using the R-hat statistic, which was at 1.0 for all fixed effects.

### Results

**Figure 5.**
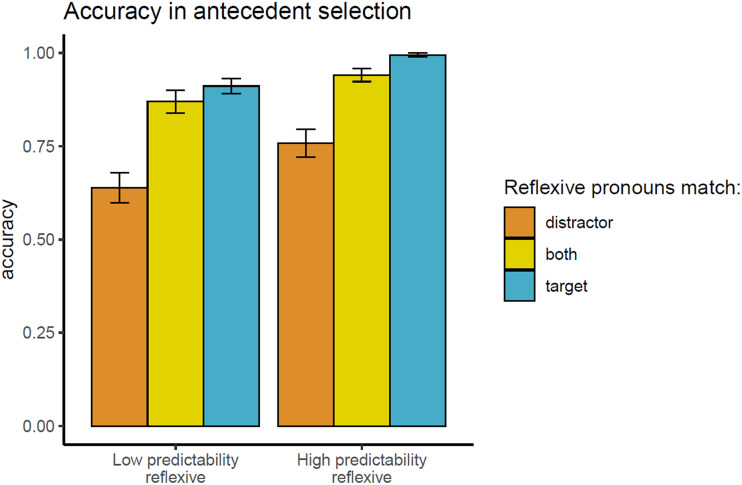
Accuracy results in Experiment 3. Mean rates of picking the subject noun as the antecedent of the reflexive pronoun, across experimental conditions.

Accuracy rates in the final comprehension task are presented in Figure 5. The results of the different models are summarized in Table 6.

**Table 6.**
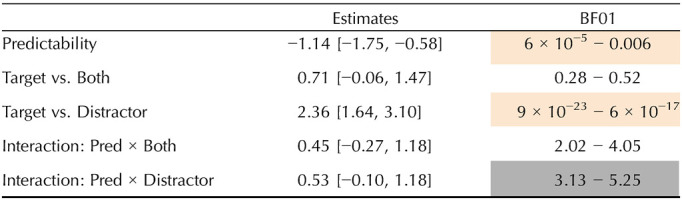
Results of Experiment 3. Mean and 95% credible interval (on the log-odd scale) of the posterior distribution for fixed effects (under the weakly informative priors set), as well as the range of BF evidence (across prior sets) for the fixed effects. Moderate evidence for the null is shaded gray. Strong or extreme evidence for an effect is shaded orange.

The results of the BF analysis suggest strong or extreme evidence for an attraction effect, such that accuracy is lower in distractor-match conditions relative to target-match ones (both-match and target-match conditions). However, we observe only anecdotal evidence for a contrast between grammatical sentences (both-match vs. target-match conditions). The results also suggest strong or extreme evidence for an effect of predictability at the reflexive, such that unpredictable reflexives (following anti-reflexive verbs) trigger lower accuracy than predictable ones (following pro-reflexive verbs). Lastly, we observe moderate evidence against an interaction of predictability with attraction.

### Discussion

Experiment 3 suggests partial evidence for attraction and against an interaction of this attraction with predictability. As was observed in Experiments 1–2, the predictability of the reflexive anaphor did not have a strengthening effect on the size of attraction. However, the evidence against interaction is weaker in comparison to the BF analysis results in Experiments 1–2, and the current measure for attraction could be confounded with guessing strategies.

In the current experiment, we found strong evidence for a higher association of the distractor with the reflexive in the ungrammatical attraction conditions. This result was obtained even though these conditions reflect a 1-feature match, which in Experiment 1 resulted in merely weak or inconclusive evidence. This could reflect increased power for the detection of attraction in accuracy measures relative to RT measures. However, another plausible explanation for this result is a strategy deployed by participants due to the format of the experiment. Participants may be uncertain about how ungrammatical utterances should be interpreted. When presented with a forced choice between two alternatives for ungrammatical sentences, participants may be more likely to resort to guessing strategies (e.g., random choice or a recency bias). This could inflate the effects associated with real-time dependency formation. In that sense, increased error rates in the (ungrammatical) distractor-match condition might reflect something other than the attraction mechanisms that generate the effect in incremental Maze RT measures.

Grammatical sentences in which the distractor matched the reflexive as well (both-match conditions) show decreased accuracy numerically, but we failed to detect evidence for/against such an effect. Namely, we could not find conclusive evidence for attraction when guessing strategies were no more likely to arise than in the basic target-match condition. In these conditions, we also failed to detect evidence for/against the interaction of predictability with the attraction contrast.

## GENERAL DISCUSSION

In this study, we set out to test the role of prediction errors in retrieval interference. We approached this from the perspective of reflexive attraction. We tested whether reflexive attraction depends on the strength of prediction for a reflexive, under the assumption that predictability of the retrieval site (the reflexive) encourages predictive dependency formation and commitment to the feature of the reflexive anaphor even after the agreement controller (the subject). This test examines whether attraction arises as part of a secondary retrieval process arising only when predictive dependency formation fails (Lago et al., 2015; Parker & Phillips, 2017; Wagers et al., 2009). Across three experiments, we observe evidence against this generalized version of the error-driven account of retrieval interference.

In Experiment 1, single-feature attraction was not observed even when a reflexive pronoun was predictable. This suggests that interference, such as attraction, is not automatically triggered when the form of the dependent in the sentence contrasts with the prediction form. In addition, this suggests that the robustness of reflexives against attraction (relative to the verb’s susceptibility) cannot be attributed to their low predictability (contra Parker & Phillips, 2017). In Experiments 2 and 3, we attempted two methods of eliciting interference for reflexives: Making the mismatch between the target and the reflexive more prominent using a two-feature design (Experiment 2) and probing the final comprehension of such attraction sentences (Experiment 3). In both cases, even though agreement attraction was detected, it still did not interact with the predictability of the reflexive. This further validates the finding that interference is not modulated by the predictability of the retrieval site and the conclusion that it is therefore part of routine parsing procedures, rather than of a special repair mechanism.

To the extent that frequency-based lexical expectations arising after the agreement controller lead to predictive computation of formal agreement, our study also yielded another, perhaps less obvious type of evidence against this general version of the Error-driven Retrieval proposal. The cost of ungrammaticality we observed in Experiments 1–2 did not interact with predictability, even though both predictability and grammaticality independently impacted RTs. In Experiment 1, we observed extreme evidence for a null interaction between predictability and grammaticality, and, in Experiment 2, evidence for this null interaction was moderate to strong. This suggests that the detection of ungrammaticality is not accompanied by a more prominent prediction error when the retrieval site is overall more predictable. Therefore, this finding too provides evidence against the existence of a unique prediction error signal associated with more predictable dependents, the signal that is hypothesized to trigger retrieval interference under the Error-driven Retrieval proposal. If ungrammaticality is detected similarly for both predictable and surprising ungrammatical elements, it follows that special retrieval procedures cannot be triggered in environments where the retrieval site is predictable.

### From Frequency-Based Prediction to Predictive Agreement Computation

While the findings are consistent across the different experiments, a crucial distinction has to be made, which might limit the scope of the findings. The error-driven retrieval hypothesis is operationalized in this study in a way that is somewhat different from how it has been formulated in the literature. The Error-driven Retrieval model debates the consequences of a specific type of predictability—predicting agreement features as part of a forward-looking dependency initiated by the agreement controller (Parker & Phillips, 2017; Wagers et al., 2009). We currently do not know of a way to directly manipulate the prediction of agreement features at the stage of presenting the subject. Therefore, our study manipulated the expectancy of a reflexive given a particular verb. We have treated this as a proxy for whether the parser predicted a syntactic (binding) dependency, including the prediction of gender features. However, the contextual surprisal of a reflexive is not the same theoretical construct as predictive dependency formation. In that sense, the current study possibly tests a weaker version of the Error-driven Retrieval model.

Still, we believe that the expectancy of a reflexive given a particular verb and the predictive formation of a dependency involving agreement features are intertwined. The likelihood of a dependency sets the motivation for executing a forward-looking dependency before encountering the second dependent. Therefore, manipulating the lexical predictability of a reflexive should also modulate the extent to which a binding dependency is predicted. Moreover, based on findings of the availability of feature specification in lexical prediction (Ito et al., 2020; Martin et al., 2018; Wicha et al., 2003), we propose that if a reflexive is highly predictable, its lexical prediction should also specify grammatical features like gender. Thus, our manipulation should be able to target the relevant prediction error associated with a mismatch between an early agreement computation and features of the dependent appearing in the input. Thus, while it is important to make the underlying assumptions explicit, we believe that they are theoretically warranted and license conclusions as to a general version of the Error-driven Retrieval model.

### Alternative Construals of the Error-Driven Retrieval Proposal

If one does not accept the premise that lexical prediction of the reflexive can involve predictive computation of agreement features, our study can be taken as an appraisal of Tung and Brennan’s (2023) finding that lexical predictability by itself (rather than as manipulation of the predictive agreement computation) modulates susceptibility to interference. Tung and Brennan (2023) present an ERP study on nominal ellipsis in Mandarin Chinese, as in (5). Nominal ellipsis is not a generally predictable dependency. Yet it could be predicted following the presence of “also” in these sentences. The ‘High Expectation’ verb *chuanle* (“*wore”*) predicts a type of object that is compatible with the nominal ellipsis in context, whereas the ‘Low Expectation’ verb *daile* (“brought”) does not. The lexical expectations triggered by the verb correspondingly raise the probability of the correct classifier: Comprehenders should predict the contextually relevant form of the classifier at higher rates in the High Expectation condition, and therefore should experience a prediction error at the ungrammatical classifier *yiben*.


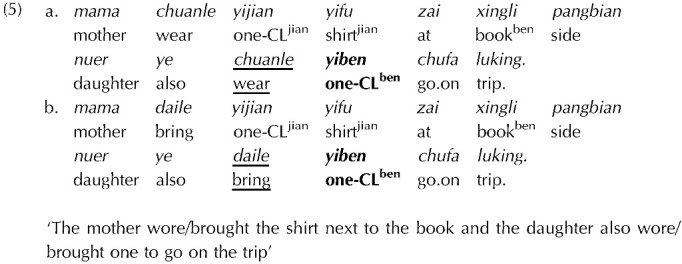


Tung and Brennan found evidence for facilitatory interference (as indexed by the P600 component in ERP measures), only in their High Expectation conditions. They interpret this result as compatible with a ‘cue preference-by-predictability’ hypothesis. On this hypothesis, an unpredicted dependency causes comprehenders to upweight structural cues. This develops the proposal by Parker and Phillips (2017) and extends it to the lexical predictability of the retrieval probe. On this view, intrusion is only expected for predictable ungrammatical dependencies, since upweighting structural cues for low-predictability dependencies should render them immune to retrieval interference.

Our findings suggest a challenge to Tung and Brennan’s idea that lexical predictability of the retrieval probe modulates interference. In our study, we exhibit reflexive anaphors to resist attraction (Experiment 1) even when they are predictable, and can be vulnerable to attraction even when they are unpredictable (Experiment 2). Thus, our findings could be taken to pose a challenge for the generalized version of the Error-driven Retrieval model promoted by Tung and Brennan, rather than the proposals delineated originally for agreement and reflexive attraction by Parker and Phillips (2017) and Wagers et al. (2009).

The contrast between our findings and those of Tung and Brennan (2023) could arise from another factor that Tung and Brennan (2023)’s manipulation affected. Their design manipulated the predictability of the target noun in its original position (in the first conjunct). This could, in principle, have an effect on susceptibility to interference. Alternatively, the contrast between the studies could be attributed to differences in the dependency type, sensitivity of the dependent measure, or strength of the predictability manipulation.

### Implications for the Grammaticality Asymmetry

Our findings provide evidence against a general version of the Error-driven Retrieval proposal as an account of the grammaticality asymmetry. However, the fact that attraction most often affects only ungrammatical sentences cannot be disputed. How then does this asymmetry arise? Several proposals are available in the literature that do not depend on the trade-off with predictability or on a repair processing stage: cue combinatorics (Wagers et al., 2009), response bias (Hammerly et al., 2019), and joint contribution of encoding and retrieval interference (Yadav et al., 2023).

Wagers et al. (2009) originally articulated two related access models that used cue-based retrieval to compute the agreement relation at the verb. One was the Error-driven Retrieval model, discussed so far. The other model assumed that retrieval happens in all sentences, grammatical and ungrammatical. They proposed to derive the grammaticality asymmetry based on cue combinatorics. The proposal treats activation not as a linear function of discrete and independent cue-matching, but as a nonlinear function of the overall distance between the retrieval cues and the item’s features. For example, Nairne (2006), following Shepard (1987), takes cue matching to be an exponential function of the distance between the retrieval cue and the memory item.

The cue-combinatorics solution to the grammaticality asymmetry is as follows. In grammatical strings, the structurally licensed subject will always match all retrieval cues: it will be a unique, full match. If matching multiple cues can yield activation that is greater than the simple additive activation of each cue (e.g., given an exponential link function, or multiplicative effect between cues), a single full match will tend to overwhelm a partial match. This favors the retrieval of the licensed subject in grammatical sentences. However, it allows the attractor to intrude frequently in ungrammatical sentences. In ungrammatical strings, no encoding will be a full match: the structurally-licensed structure and the attractor will both be non-unique, partial matches. In this case, multiple partial matches will be equidistant from one another and thus still compete for retrieval. Overall, some balance between cue combinatorics and retrieval threshold can therefore block the effect of the distractor in grammatical sentences while still allowing access to partial matches in the absence of a full match (thus licensing attraction in ungrammatical sentences).

Alternatively, accounts of attraction that focus on encoding errors and representational distortion (as explained in section Interference In Dependency Formation) can provide an additional mechanism that offsets the predicted ungrammaticality illusion (Hammerly et al., 2019; Yadav et al., 2023). Hammerly et al. (2019) suggest that comprehenders have a strong expectation for grammaticality. Since more often than not, sentences unfold grammatically, comprehenders are more likely to conclude that an ungrammatical dependency is grammatical and are less likely to conclude that a grammatical dependency is ungrammatical. This is a form of response bias. Coupling this bias with an attraction based on encoding errors can capture the grammaticality asymmetry: the distortion of the subject’s features surfaces in ungrammatical sentences, while the bias makes comprehenders less sensitive to variations in grammatical sentences.

Yadav et al. (2023) proposed a hybrid account that takes advantage of the fact that Cue-Based Retrieval and encoding approaches have opposite predictions for grammatical sentences. Under Cue-Based Retrieval, a distractor matching the target should slow down access to the target. This retrieval approach, therefore, predicts longer reading times in (6a) compared to (6b). On the other hand, if mismatching features of the distractor distort the representation of the target, comprehenders are expected to occasionally perceive sentences like (6b) as ungrammatical. Therefore, the encoding approach predicts a contrast of the opposite direction, whereby grammatical sentences with a mismatching distractor (6b) are read slower than unambiguously grammatical ones (6a). If both retrieval and encoding types of interference arise, the two effects can offset each other. Yadav et al. (2023) propose that this can capture the grammatical asymmetry: For ungrammatical sentences, both factors push in the same direction, aggravating the misperception of the same configuration as grammatical. In the processing of grammatical sentences, the two push in diverging directions and cancel out the behavioral effects.

(6)  a. The **door** to the laboratory **was** accidentally left unlocked
    b. The **door** to the laboratories **was** accidentally left unlocked

Our findings cannot distinguish between these alternatives and are compatible with all three accounts of the grammaticality asymmetry. They only preclude an account in terms of Error-driven Retrieval, which predicts an interaction between predictability on the one hand, and attraction (and grammaticality) on the other hand. Future research into the balance between encoding and retrieval interference, as well as into the cue combinatorics of retrieval, will be able to shed light on the remaining accounts.

### Implications for the Processing of Reflexives

Our results also have broader implications for research into how reflexive dependencies are resolved in comprehension. The word-by-word processing data in Experiments 1 and 2 are consistent with Parker and Phillips’ (2017) claim that reflexive attraction is selective and limited to configurations where the target mismatches the reflexive in multiple features. In Experiment 1, when the target antecedent mismatched the reflexive only in a single feature (gender), we saw no attraction in Maze RTs. Bayes Factor analyses revealed strong evidence in favor of the null model, i.e., no effect of attraction in Experiment 1. However, when the target mismatched the verb in two features (number and gender; Experiment 2), we saw clear evidence of attraction from structurally illicit distractors. The finding that there is no measurable interference in online processing times when the target mismatches in one feature is in line with some previous studies (Cunnings & Sturt, 2014; Dillon et al., 2013; Parker & Phillips, 2017; Sloggett, 2017; Sturt, 2003). Yet, it conflicts with recent findings of Jäger et al. (2020), which report evidence for such reflexive interference in total reading time measures in eye-tracking. The question of why reflexive dependencies exhibit attraction in some studies but not in others remains open.

Although we did not see interference with single-feature mismatched targets in our online measures, there is a very large body of experimental evidence showing such interference in verbal agreement (Dillon et al., 2013; Lago et al., 2015; Pearlmutter et al., 1999; Wagers et al., 2009). One question is why verbal agreement should differ from reflexives, such that comprehenders place a higher weight on syntactic retrieval cues for reflexives than agreement (Cunnings & Sturt, 2014; Parker & Phillips, 2017). Our results are not consistent with the idea that it is the general predictability of the agreeing element that drives the difference between verbal agreement and reflexives, since our results give evidence that susceptibility to interference is independent of predictability.

One remaining possibility is that reflexives and verbs target different levels of representation and hence use slightly different cue sets, or cue weights for accessing the subject in these different representations. Reflexive anaphors have to undergo syntactic binding to receive their reference. Gender and number features of a reflexive are thus more integral to its interpretation of anaphors than to that of verbs. Consequently, feature-matching processes with reflexives might be distinct from those involved in simple agreement relations. Syntactic processes are also involved in the integration of a verb into the sentence. However, these syntactic processes—constructing the relevant phrase structure, case assignment, and agreement—are not part of the interpretation verb. Therefore, retrieval triggered by a reflexive might target syntactic representations with mostly structure-based cues, while retrieval initiated by a verb would involve more lexical-semantic features. Our results do not directly support such a distinction, and so at this point, this is merely a speculation. We leave resolving this question to future research.

Finally, our manipulation of reflexive predictability and grammaticality, and in particular our offline interpretation findings, offer an argument against a possible noisy channel account of reflexive attraction. Noisy channel processing is a form of rational (Bayesian) inference and allows comprehenders to consider nonliteral interpretations of the input (Gibson et al., 2013). These interpretations would be chosen if they yield a more probable message, given the likelihood of speaker errors and prior probabilities over different interpretations. Reflexives in general are infrequent, and therefore, a reflexive interpretation would normally have low prior probability. This means that comprehenders might assume that a reflexive form like *herself* was erroneously produced instead of a pronominal like *her*, which has a higher prior probability. This might entail sensitivity to gender features of sentential antecedents other than the subject, namely attraction. However, if the prior probability of alternative interpretations plays into comprehenders’ interpretation of the reflexive, then we would expect that the more predictable a reflexive is, the less likely the comprehender is to interpret it (non-veridically) as a pronoun (i.e., with the distractor as a possible referent). This prediction is not borne out: Attraction was just as prominent when a reflexive pronoun was predictable or unpredictable given the verb. We take this observation to suggest that the prior probability of different interpretations (e.g., reflexive versus non-reflexive events) does not modulate the rate of grammatically illicit interpretations of our stimuli, which would have been expected from a noisy-channel perspective. This suggests a puzzle that might drive future research, namely, why are interpretations for ungrammatical reflexive dependencies not sensitive to the prior probability of possible alternative messages, as might be expected (Gibson et al., 2013; Poliak et al., 2024; Ryskin et al., 2021).

### The Relation Between Predictability and Retrieval in Previous Studies

Both our findings and the Error-driven Retrieval model stand in contrast to other experimental investigations of the relationship between predictability and retrieval (Campanelli et al., 2018; Husain et al., 2014; Schoknecht et al., 2022). These previous studies proposed that the predictability of words triggering the retrieval can attenuate retrieval interference, based on reading times (Campanelli et al., 2018; Husain et al., 2014) and ERP data (Schoknecht et al., 2022). Campanelli and colleagues, for example, crossed predictability and interference manipulations in a self-paced reading task and found evidence that retrieval interference was *reduced* for more predictable verbs. Such attenuation would arise if predictability lets comprehenders pre-activate potentially relevant items in memory in advance of the critical retrieval event (see also Lewis et al., 2006; Vasishth & Lewis, 2006). Increasing the target’s activation in this way should lead to less interference from any distractors in memory. Campanelli and colleagues propose a mechanism whereby highly predictable elements can be preactivated ahead of time, allowing comprehenders to gather evidence about the dependencies in the input before receipt of the verb; This in turn reduces the need for backwards-looking, interference-prone retrieval operations.

Since we failed to find that the predictability of a reflexive did not reduce the amount of retrieval interference observed, our results seem to conflict with this claim. This view on the prediction-retrieval tradeoff also predicts effects that are exactly the opposite of those predicted by the Error-driven Retrieval model: The more predictable a dependency is, the less vulnerable it would be to retrieval interference. However, there are several major differences between the view of the prediction-retrieval trade-off in Campanelli et al. and the focus of our investigation (and the Error-driven Retrieval model). First, different senses of prediction are targeted here and in those previous studies. The Error-driven Retrieval model focuses on the prediction of syntactic dependencies, while the prediction-retrieval tradeoff discussed by Campanelli et al. (2018) targets lexical prediction. Second, while our study and the Error-driven Retrieval Model target vulnerability to interference in the face of ungrammatical input, the other studies mentioned here focus on prediction and interference in grammatical input. It is possible that additional predictive power mitigates reliance on retrieval in grammatical dependencies (reducing so-called inhibitory interference), but does not reflect on the likelihood of incorrectly perceiving an ungrammatical dependency as acceptable (so-called facilitatory interference). Thus, the findings of our study and previous findings (Campanelli et al., 2018; Husain et al., 2014; Schoknecht et al., 2022) might not be as contradictory as they seem at first glance.

Lastly, minor design differences could affect the amount of predictive preactivation. These differences include how constraining linguistic contexts are those counted as high predictability ones (Cloze probability), the time available to generate a prediction (as compared to Campanelli et al., 2018 and Husain et al., 2014), and effects of temporary referential ambiguity (as compared to Schoknecht et al., 2022). Possible differences in predictive preactivation make it difficult to compare results across the studies. More work is needed to determine the extent to which our results conflict with the predictive mechanism advocated by Campanelli and colleagues.

Additional studies have recently argued for an interaction between predictability and retrieval interference (Tung & Brennan, 2023; Xu & Futrell, 2025). These studies manipulate the predictability of the target word, that is, the earlier dependent, and find that this modulates the vulnerability to attraction and facilitatory interference. These findings do not conflict with our proposal as they concern the predictability of the left dependent rather than that of the retrieval site. More specifically, both studies mentioned above find that low predictability targets are less vulnerable to interference. We believe that such findings do not require an Error-driven Retrieval framing. Effects associated with the predictability of the target could be attributed to the sensitivity of memory encoding processes to novelty/informativity (Lewandowsky et al., 2008; Xu & Futrell, 2025), which precede the retrieval process.

### Limitations and Future Research

The findings of this study should be further extended in the future to verbal agreement attraction. While it is not trivial to manipulate the predictability of verbal agreement, this extension is important for establishing our conclusions. The evidence for a routine retrieval mechanism is currently partially limited by two main drawbacks of reflexive attraction. First, reflexives are of relatively low probability even when they are predictable in context. While we tried to maximize the predictability of reflexive pronouns, their occurrence was still far from certain. Verbs, on the contrary, appear in any sentence, and number marking appears on many of those in English (present tense of all aspects and past progressive). Therefore, it is possible, in principle, that the interaction between predictability and interference occurs only for highly predictable dependencies. Our reflexive predictability manipulation was successful in modulating comprehenders’ predictions for a reflexive. Such a prediction for a reflexive should be accompanied by a predictive dependency formation (prediction of specific features). Still, we cannot rule out the possibility that no prediction of a syntactic dependency nor a commitment to a predicted gender features of the reflexive is made, even in cases where a reflexive anaphor is relatively predictable. Thus, further research is needed to corroborate that our findings also extend to cases with a stronger contrast between predictable and unpredictable dependencies. Only this additional test would unambiguously target the active dependency formation process implicated in the Error-driven Retrieval model.

Another possible concern is that the processing of reflexives might use retrieval mechanisms that are qualitatively distinct from those that are used for other syntactic dependencies like verbal agreement (e.g., Dillon, 2011). If reflexives use qualitatively distinct retrieval processes, our current findings may not be straightforwardly extendable to other cases of interference. Nonetheless, we believe that our findings still shed light on the Error-driven Retrieval proposal for two important reasons: (i) our findings counter in broad lines the hypothesis put forward by Parker and Phillips (2017) that the distinction between reflexive attraction and verbal agreement attraction can captured in terms of prediction error and the triggering of error-prone repair mechanisms; (ii) our findings provide evidence against an assumption of the Error-driven Retrieval proposal, in its generalized version, according to which perceiving an ungrammatical constituent involves additional prediction error when that constituent is lexically predictable.

## CONCLUSIONS

Forming a dependency between distant linguistic constituents is a crucial task for sentence comprehension. Much work has revealed that carrying linguistic information across intervening input arises through a combination of forward-looking (predictive) processes and retroactive memory retrieval, where interference often arises. Some proposals in the sentence processing literature have questioned the extent to which normal processing relies on such interference-prone memory retrieval (Lago et al., 2015; Wagers et al., 2009). Here, we present some evidence that interference is part of routine processing operations, rather than a by-product of an ad-hoc repair strategy of the parser. We find, in the context of reflexive attraction, that attraction effects are not modulated by prediction failure, and therefore we propose that they are not likely to reflect a secondary processing operation which is only recruited when this ‘primary’ (forward-looking) dependency formation fails. We suggest that memory retrieval, including interference-prone retrieval, arises as a primary, rather than a secondary, dependency formation strategy.

## ACKNOWLEDGMENTS

We thank Travis Heller (University of California, Santa Cruz) for collecting predictability norms and Amanda Doucette (University of Massachusetts Amherst) for constructing the initial set of stimuli.

## FUNDING INFORMATION

The research was supported by the NSF/US-Israel Binational Science Foundation (NSF-BSF 2146798 to BD, UMass Amherst) and the National Science Foundation (NSF BCS-1941485 to BD, UMass Amherst). During the course of this project, MK was also supported by the Azriely Early Career Faculty Fellowship, and MW was supported by the National Science Foundation (NSF BCS-2019804 to UC Santa Cruz).

## AUTHOR CONTRIBUTIONS

Maayan Keshev: Conceptualization; Formal analysis; Investigation; Methodology; Project administration; Validation; Visualization; Writing – original draft; Writing – review & editing. Kaiva Hinkle: Investigation; Methodology; Writing – original draft. Matthew Wagers: Conceptualization; Methodology; Writing – original draft; Writing – review & editing. Brian Dillon: Conceptualization; Funding acquisition; Methodology; Supervision; Writing – original draft; Writing – review & editing.

## DATA AVAILABILITY STATEMENT

The study was not pre-registered. Data, materials, and analysis code associated with this study (Experiments 1−3) are available through OSF at osf.io/2wc46/?view_only=815da1d82499437b9413494812124da7.

## Figures and Tables

**Figure A1.**
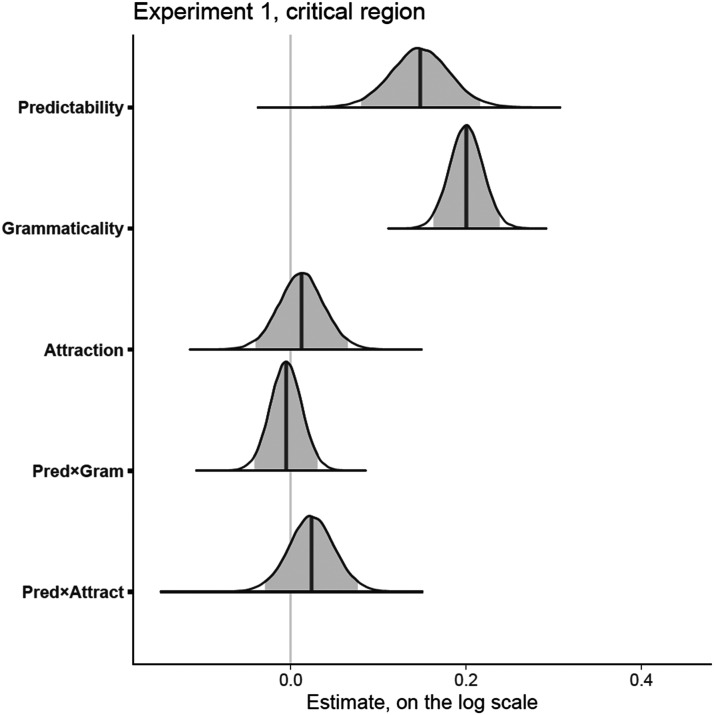
Results of Experiment 1 at the critical region. Posterior distribution of main effects and interactions (under weakly informative priors) on the log scale.

**Figure A2.**
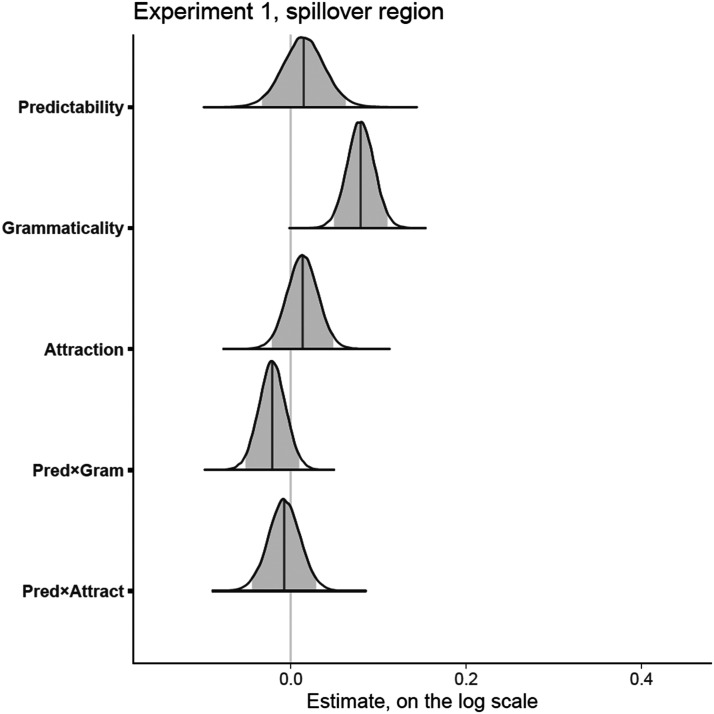
Results of Experiment 1 at the spillover region. Posterior distribution of main effects and interactions (under weakly informative priors) on the log scale.

**Figure A3.**
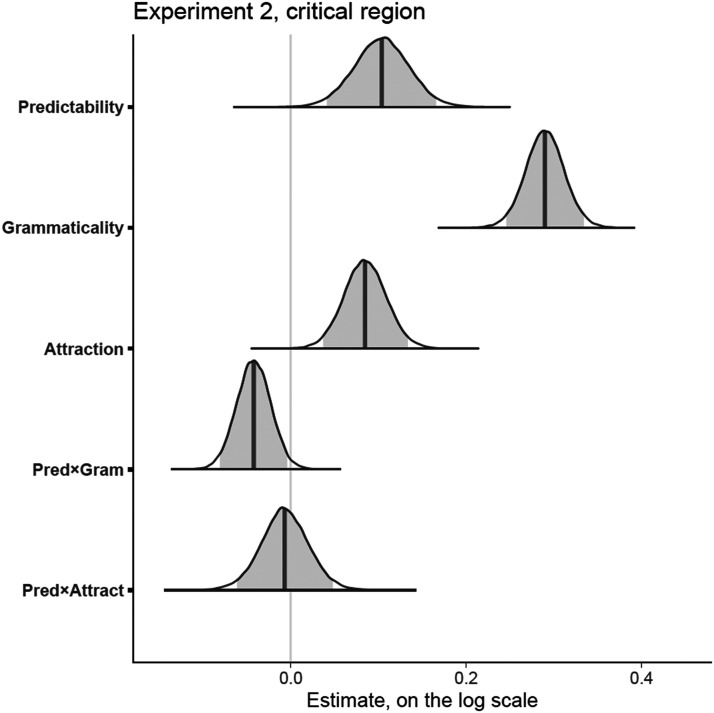
Results of Experiment 2 at the critical region. Posterior distribution of main effects and interactions (under weakly informative priors) on the log scale.

**Figure A4.**
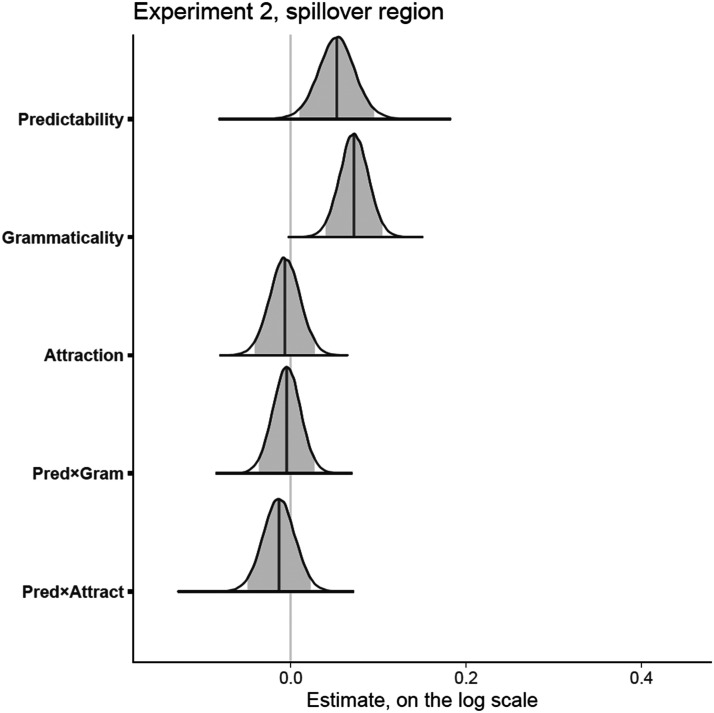
Results of Experiment 2 at the spillover region. Posterior distribution of main effects and interactions (under weakly informative priors) on the log scale.

**Figure C1.**
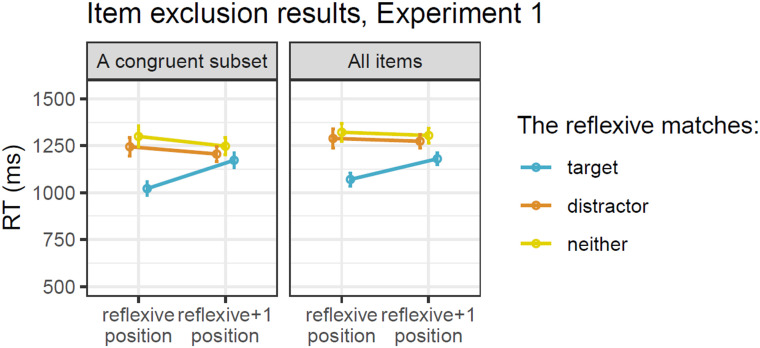
Results of the low-predictability conditions before and after item exclusion, Experiment 1.

**Figure C2.**
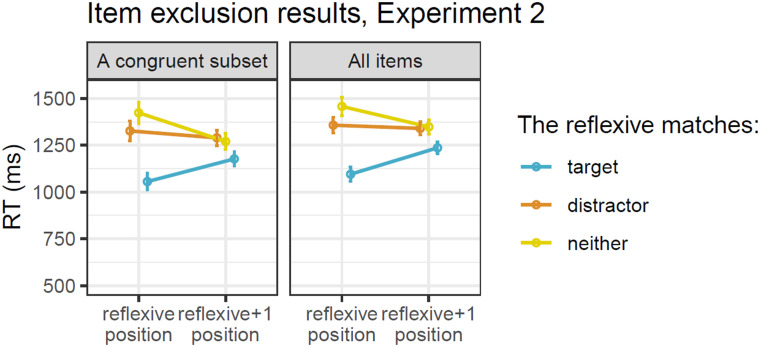
Results of the low-predictability conditions before and after item exclusion, Experiment 2.

## APPENDIX B: POST-HOC RE-CODING OF THE PREDICTABILITY FACTOR AS CONTINUOUS

Here we report an additional analysis of Experiments 1–2. In this analysis, we present the predictability of a reflexive as a continuous factor rather than a binary low vs. high predictability distinction. We operationalize a reflexive’s predictability as the probability of a reflexive pronoun given the verb, on the log scale, in line with Surprisal (Levy, 2008). To determine this continuous measure of predictability, we used the COCA frequency information that was extracted in the construction of the items (see subsection Materials).

As in the main analyses reported in the paper, we analyzed RT using Bayesian hierarchical models with a lognormal link function. RTs at the critical reflexive region and on the following word (the spillover region) were analyzed in separate models. The model included weakly informative priors: a standard normal distribution, N(0, 1), as the prior for fixed effects and the standard deviation parameters, a wide normal prior of N(0, 10) for the intercept, and the LKJ prior for correlation matrices of random effects. Four Monte Carlo Markov Chains of 10,000 iterations each were sampled from the posterior distribution. The first 4,000 samples of each chain were discarded as a warm-up. Convergence was checked using the R-hat statistic, which was at 1.0 for all fixed effects.

Tables B1 and B2 summarize the posterior distributions obtained from the continuous Surprisal model of Experiment 1 and Experiment 2 (respectively). For convenience, the tables also repeat the results from the models of the main analysis. These corresponding models (which also appear in Tables 2 and 4, in the main text) had a binary low/high predictability factor sum coded but included the same prior settings.

**Table B1.** Results of Experiment 1 under the continuous Surprisal measure of a reflexive’s predictability. Results under the categorical division to low vs. high predictability environments are repeated for ease of comparison. Mean and 95% credible interval of the posterior distribution for fixed effects (under the weakly informative priors set), on the millisecond scale.

	Reflexive region		Spillover region	
	Categorical (low/high) predictability	**Continuous Surprisal**	Categorical (low/high) predictability	**Continuous Surprisal**
Predictability	149 [81, 219]	25 [19, 34]	16 [−39, 72]	2 [−3, 8]
Grammaticality	196 [160, 232]	174 [121, 227]	91 [56, 125]	118 [57, 179]
Attraction	15 [−42, 72]	−34 [−109, 42]	16 [−26, 58]	39 [−36, 113]
Interaction: Pred × Gram	10 [−25, 44]	3 [−8, 14]	−23 [−58, 11]	−6 [−16, 4]
Interaction: Pred × Attract	27 [−31, 84]	8 [−7, 23]	−9 [−53, 34]	−5 [−16, 7]

**Table B2.** Results of Experiment 2 under the continuous Surprisal measure of a reflexive’s predictability. Results under the categorical division to low vs. high predictability environments are repeated for ease of comparison. Mean and 95% credible interval of the posterior distribution for fixed effects (under the weakly informative priors set), on the millisecond scale.

	Reflexive region		Spillover region	
	Categorical (low/high) predictability	**Continuous Surprisal**	Categorical (low/high) predictability	**Continuous Surprisal**
Predictability	108 [39, 177]	19 [12, 26]	62 [12, 112]	9 [4, 15]
Grammaticality	302 [257, 346]	320 [254, 385]	84 [46, 121]	85 [23, 144]
Attraction	102 [44, 161]	102 [15, 189]	−8 [−50, 33]	−2 [−78, 73]
Interaction: Pred × Gram	−27 [−66, 11]	−3 [−15, 8]	−3 [−40, 33]	0 [−11, 11]
Interaction: Pred × Attract	−4 [−70, 62]	0 [−16, 16]	−16 [−60, 27]	−1 [−14, 12]

Comparing the two operationalizations of a reflexive’s predictability, we observe overall similarity with some minor differences. The most noticeable difference between the models is the effect size associated with the predictability factor. In the continuous Surprisal model, predictability has a much smaller effect relative to the binary low/high predictability model. Yet, even in the continuous Surprisal model, the 95% credible interval of the predictability effect excludes zero. This indicates that a predictability effect is likely to be smaller than indicated by the main model, though it is still not very likely to be null. The decrease in effect size is to be expected: A binary categorization forces a sharp contrast, while in the continuous Surprisal measure, the effect is more gradient.

In contrast to the predictability factor, the effect size of the grammaticality factor remains consistent across the two model types. Estimates of this effect remain high, and the 95% credible intervals fall far from zero. The same is true for the attraction factor in the critical region of Experiment 2. This effect was pronounced and reliably distinct from zero on the main analysis, and remains so in the continuous Surprisal model. The attraction effect in the critical region of Experiment 1 might seem to be distinct from that obtained in the main analysis. The directionality of this effect, as indicated by the posterior distribution’s mean, has shifted. Still, we do not consider this an informative diversion between the analyses, as the credible intervals of the two analyses highly overlap, and both include zero. This suggests that the shift of the mean, from one side of the zero point to the other, is not likely to represent very different effect estimates.

Other effects are centered near zero, under both model types. Critically, even under the continuous Surprisal analysis, the interaction terms have credible intervals that include zero, in both regions of both experiments. The credible intervals that were obtained in this analysis highly overlap those obtained in the binary low/high predictability model. This validates the results from our main analysis.

Overall, we conclude that an analysis with a reflexive’s predictability coded as continuous Surprisal yields results that are highly compatible with those obtained in our main analyses, where predictability was binned into a binary contrast of low vs. high predictability.

## APPENDIX C: POST-HOC EXCLUSION OF IMPLAUSIBLE LOW-PREDICTABILITY ITEMS

After collecting the data and reviewing our experimental items again, we wanted to examine whether the results in the low predictability conditions were biased by a subset of the items in which a reflexive pronoun might be incongruent, rather than just unlikely. When removing this subset of items from the low predictability conditions, the pattern remained the same.
